# IEyeGASE: An Intelligent Eye Gaze-Based Assessment System for Deeper Insights into Learner Performance

**DOI:** 10.3390/s21206783

**Published:** 2021-10-13

**Authors:** Chandrika Kamath Ramachandra, Amudha Joseph

**Affiliations:** Department of Computer Science and Engineering, Amrita School of Engineering, Amrita Vishwa Vidyapeetham, Bengaluru 560047, India; j_amudha@blr.amrita.edu

**Keywords:** gaze detection, human-computer interaction, user behavior understanding

## Abstract

In the current education environment, learning takes place outside the physical classroom, and tutors need to determine whether learners are absorbing the content delivered to them. Online assessment has become a viable option for tutors to establish the achievement of course learning outcomes by learners. It provides real-time progress and immediate results; however, it has challenges in quantifying learner aspects like wavering behavior, confidence level, knowledge acquired, quickness in completing the task, task engagement, inattentional blindness to critical information, etc. An intelligent eye gaze-based assessment system called IEyeGASE is developed to measure insights into these behavioral aspects of learners. The system can be integrated into the existing online assessment system and help tutors re-calibrate learning goals and provide necessary corrective actions.

## 1. Introduction

Learning assessment is a fundamental feedback mechanism that allows the stakeholders to understand what is being learned and where the learning resources need to be focused. Assessment can take different modalities based on the purpose. It can either be summative or formative [1,2]. When an assessment is a part of the teaching process, carried out in a classroom and involves evaluator observation, feedback, homework, etc., it is called a formative assessment. It is relevant to understand the learning needs of learners and to adjust the instruction accordingly. Summative assessments determine to what extent the learners have achieved the learning goals and acquired the critical knowledge and skills related to educational content. They are usually conducted at the end of an instructional unit. Summative and formative assessments can be either objective or subjective. Subjective is a form of questioning where there is more than one way of expressing the answer. Objective assessment includes true/false, multiple-choice, and matching questions.

Multiple-choice questions have a strong association with assessing lower-order cognition, such as recall of concepts, and hence, they are popularly used in online assessment in higher education. The education sector is slowly replacing the traditional methods of pen and paper assessment with online assessment. The online evaluation produces quick results on the learners’ progress. It provides insight into how they are doing and what areas of learning require attention [3,4]. Educators use various tools like Google Form, Socrative [5], Mentimeter [6], etc., for online assessment. Most of these tools help create multiple choice-based assessments and provide quick feedback on the learner’s performance. However, the feedback is based on the score, the option chosen, time spends on each question, response time, etc. It provides whether the learner has memorized the concepts or not. Well written multiple-choice-based assessment can also evaluate higher-order thinking such as application, creativity and analytics skills. Intelligent tutoring systems by [7] predicts learner performance via summative and formative assessments and also predict learners at risk of failure in the final evaluation. However, these do not provide deeper insights into how the learner’s answered the questions, what was perceived, was it a mere guess, was there a state of confusion, what factors lead to answering the correct option or incorrect option etc. An objective assessment that can quantify the cognitive parameters of learners can explain the factors that lead to correct or incorrect answers. Cognitive parameters provides insights into the mental process of gaining knowledge like engagement in the task, the order and time for processing information, time spent on crucial details in the task, etc of a learner. Depending upon the skill set to measure, researchers use various tests like the Predictive Index Learning Indicator test [8], McQuaig Mental Agility Test [9], Progress test [10] and technologies like EEG, eye tracking etc [11,12,13,14] to measure the cognitive parameters. Progress test is one of the assessment methods of the cognition domain used to access students on topics from medical disciplines. In this research work, we use eye tracking technology to develop an intelligent system that can quantify the cognitive parameters and provide feedback on the learner performance in an objective-based online assessment for programming related courses.

Eye tracking is a sensor technology that lets a computer detect the attention and focus of the learner. The technology investigates the visual attention based on the eye–mind hypothesis [15]. According to the hypothesis, there is a close relationship between eye gaze and attention while performing any task involving information processing. It has been applied in various research areas such as reading [16,17,18,19,20], comprehension [21,22], information processing [18,23], human-computer interactions [24,25], skill identification [26,27], problem solving [28,29], e-learning and learner evaluation [3,30,31,32,33,34,35]. These studies provide an insight into the cognitive strategies of individuals, amount of attention, difficulties in reading, and their learning process. These research studies prove that there is a link between eye-gaze patterns and the cognitive process. Eye tracking technology is best suited for multiple-choice (MC) assessment. It allows recording the learner’s attention distribution while solving a task without placing any extra load on the learner’s working memory. Moreover, the recordings are objective and provide data, both temporal and spatial, that can reveal unconscious cognitive events like decision-making behaviour that are not accessible from external observations. To investigate the decision-making behavior of learners in an MC assessment, we applied Bloom’s taxonomy [36]. Bloom’s Taxonomy is a hierarchical way of classifying levels of thinking and applied by universities to design course objectives. It has six levels of thinking - Remember, Understand, Apply, Analyze, Evaluate and Create. Bloom’s Taxonomy is explained in detail in Section 4.1. In this research work, we focused on the basic level of thinking called the remember, where the learner must recall the information from the long-term memory. It will help us in probing questions like why the learner cannot perform, whether he hasn’t understood the concept/learned the concept, whether he overlooked the question, whether the multiple-choice question is generally designed with confusing options and make the learner struggle to perform? Eye tracking data will provide insight into these aspects and helps to redefine the specific goals and objectives based on the feedback and recommendations.

In this research work, we developed an intelligent system called “Intelligent Eye Gaze learner AsSEssment System” (IEyeGASE) to analyze the eye gaze of the learners taking an online assessment and map to cognitive parameters to provide deeper insights into their performance. The IEyeGASE system can be integrated with an online assessment system to quantify the learners’ performance based on engagement in the task, attention to critical information in the task, wavering behaviour, knowledge acquisition and confusion between various options.

The following section will discuss relevant works in educational science and eye tracking research for computer-based testing and then the proposed methodology for developing the intelligent eye gaze-based learner assessment system. This section will discuss the methodology, various hypothesis and cognitive parameters like wavering behaviour, engagement in the task, inattentional blindness etc, that will provide insights into a learner’s performance. In the following section, the materials used for displaying MCQs, eye tracking study apparatus, and participants’ details followed by results and a comparison of the intelligent system with a traditional pen and paper approach and online assessment platform.

## 2. Related Works

Despite the best efforts from the academicians, learners struggle with programming activities particularly in a freshman course [37]. Various analytical methods [38,39] and e-learning platforms [30,32,40] are used to improve the learning experience of learners. All these research works focused on learning design and its evaluation but very few works [3,4,28,29,31,34,35,41,42] focused on learner’s decision-making, lesson-specific skills and problem-solving skills. The study [2], discussed on use of computer-based tests to support and access student learning. They investigated the effects of adapting computer-based test items according to multimedia learning guidelines. The results indicate that adapting multimedia guidelines can lower students’ difficulty, lead to more attention in questions and answers, and reduce their cognitive load.

Speech analytics were used in the traditional assessment to assess the reading proficiency of the learners [43]. Machine learning algorithms were further used to evaluate the knowledge level of learners based on the expert formulated responses [44]. Non-intrusive methods were then used to assess the learner behavior during assessment [3,4,31,34,35]. These research works used Multiple-Choice Questions (MCQs) to test the learner’s knowledge.

Eye tracking research is increasingly used in educational science in three major areas- improving the instructional design of computer-based learning and testing environment, developing expertise in visual domains, and promoting visual expertise using eye tracking modelling examples [45]. The instructional design investigates how learning a new skill or knowledge by optimally designing the learning material. Eye tracking can help understand how learners process instructional material and which processes learners should devote to achieving learning gains efficiently [46]. Eye tracking research is extensively used to develop expertise and understand how long-term memory structures influence how we see and interpret our environment [21,47,48]. Lastly, eye tracking is applied in educational science is to investigate how visual expertise can be trained with the help of instructional videos of real-world tasks that experts explain in the field. This is mostly used in guiding novice learners [49,50,51]. Our research work focuses on applying eye tracking to understand learner behaviour in a computer-based testing environment. In the following section, we discuss a few of the relevant works in this area.

Eye tracking is used to inspect the learner’s visual attention while solving multiple-choice questions in mathematical problems related to the Pythagorean theorem and related factors [34]. Percentage gaze time on the AOIs was investigated to find the most probable choices to questions. The possible choices had a higher percentage gaze time than the less probable ones. As soon as the most probable choices were recognized, a wavering behavior between the two alternatives was observed in testers. Wavering behavior was found to be less once the tester gives the first answer. Then gaze patterns were compared with previously stored patterns to predict the success of the solution. The research work inspected the way testers dealt with MCQs and provided these inputs to develop an intelligent tool to help learners learn better. In another work by [52] AOI coverage is used as a metric to compare the natural language text and source code reading behaviour of expert and novice. A two level abstraction was considered, element/word level and line level. The segmenting of stimuli into multiple levels of abstraction revealed detailed insight into the reading behaviour of novice and expert. A similar study was conducted [3] to solve the MCQs in science problems. The study shows that the successful problem solvers gazed at relevant options, and unsuccessful problem solvers struggled in understanding the problem and recognizing the most relevant factors. Percentage fixation duration was used to evaluate the attention on appropriate choices.

An eye tracking system was developed to predict a computer-based assessment performance system for physics concept questions [31]. The study investigated the eye tracking features for predicting performance of learners. The results indicate that mean fixation duration and re-reading time was more probable for predicting the correct options, whereas mean saccade time was less likely to predict the correct option. Pictorial and text representation were presented to learners. Pictorial representation was found to be more preferred by learners. The importance of gaze bias effect for decision-making process of learners in knowledge assessment was investigated in [4]. Eye gaze patterns of High Prior Knowledge (HPK) and Low Prior Knowledge (LPK) were recorded. HPK learners and LPK learners were found to have a gaze bias effect on options that are subjectively preferred, whereas HPK learners fixated more on the objectively correct option. 21 MCQs were presented to the learners, and each one has correct, attractive, and non-attractive options. Percentage gaze duration was the used for analysis of their performance. The study provided evidence of the generalizability of the gaze bias effect in the decision-making process.

In [53] authors developed an online hypothesis testing platform for experimental research in cartography and psychological diagnostics.The platform consists of various modules for creation of tasks, management of test and users and allows export of raw data for analysis. The tool can be used for collaborative tasks and can be integrated with eye tracking systems. However, this tool does not measure cognitive parameters of the users. In another research work [29], pilot study by was conducted to investigate the problem-solving behaviors of learners with different levels of expertise in three disciplines of science (biology, chemistry, and physics). The study provides enough evidence to prove that eye tracking can distinguish different levels of expertise across disciplines. In the work [54], eye movements were used to investigate the bug fixing task. The participants were grouped as experts and novices. The results indicate that programmer code reading behaviour measured using eye tracking, at line level and element level can used to differentiate the participant expertise.

The related works in eye tracking indicate that eye gaze can reveal learner behaviour during the assessment and give insight into their domain knowledge and decision-making process. Most studies use eye tracking to differentiate the learner’s expertise and understand reading behaviour and problem-solving skills. However, few studies use eye tracking technology for online evaluation and provide feedback on what factors affected the learner performance.

## 3. Intelligent Eye Gaze Learner AsSEssment System-IEyeGASE

**Figure 1:**
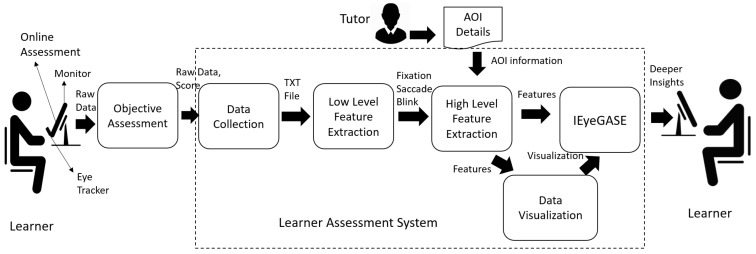
Learner Assessment System.

The related works indicate that eye tracking technology can provide insights into learners’ learning behavior and decision-making process. However, a few studies have used it to develop a system to assess the learner and provide personalized feedback.To understand the learner’s cognitive and visual behavior during the assessment and provide deeper insights into the performance of learners, we developed an intelligent learner assessment system called Intelligent Eye Gaze-based learner AseSEsment (IEyeGASE) system. The IEyeGASE system tracks the eye movements of the learners while taking an online objective assessment and provides personalized feedback on their performance in the test based on various hypothesis and cognitive parameters. An online assessment system usually provides a score to quantify the performance of the learner. The proposed learner assessment system will provide a deeper understanding of what cognitive factors influenced the learner’s performance and provide personalized feedback. Cognitive parameters like wavering behaviour, confusion, engagement in task, findability, knowledge acquisition etc., provide s with detailed insights into learners’ performance that can be used to recalibrate learning goals, and help learners take corrective actions. A personalized feedback is provided for every objective question attempted by the learner. Figure 1 represents various modules of the proposed learner assessment system. It has the following modules:

1. Objective Assessment
2. Data Collection
3. Low Level Feature Extraction
4. High Level Feature Extraction
5. Intelligent Eye gaze AsSEment system -IEyeGASE
6. Data Visualization

The objective assessment system provides an interface for the learner to interact with the learner assessment system. The data collection module collects the learner’s raw eye movements while the learner interacts with the system. The low level and high level feature extraction modules generate the features from raw eye movements. These features are mapped to various cognitive parameters and hypothesis discussed in Section 3.6. Finally, the IEyeGASE system uses these high level features to analyze learner performance and provide the personalized feedback. The different modules of the learner assessment system are discussed in detail in the following subsections.

### 3.1. Objective Assessment

The objective-based assessment module is the interface to display the learner with the objective-based assessment questions. In this research work, multiple-choice questions(MCQs) are used for assessing the performance of the learners. It is designed using the SMI Experiment suite 360° [55]. The interface displays the MCQs discussed in Section 4.2 one after the other. The learner response is manually recorded using the Think Aloud method [56], where the learner answers the question loud enough for the observer to record the information. A binary score of 1 or 0 was given to right and wrong answers, respectively.

### 3.2. Data Collection

The data collection module records the raw eye gaze data using SMI Redn Professional [57] eye tracker while the learner is being assessed. The raw sensory data consists of various fields like participant details, calibration details, calibration area, system details, timestamp, trial number, gaze position, pupil position, pupil diameter, and quality values. Gaze position is the coordinate points inside the calibration area at which the user gazed. In this study, the calibration area is the screen area. For eye gaze data analysis, the data points of interest are timestamp and gaze position represented using Raw X and Raw Y coordinates. The raw data is an IView Data File (IDF) that is further converted to a text file using an IDF converter tool provided by SMI manufactures.

### 3.3. Low Level Feature Extraction

The low level feature extraction module generates various events from the raw eye movements data. Eye movements are represented in terms of low-level features like fixations and saccades. Fixations are the fixed gazes over the informative region of interest, and saccades are rapid eye movements between fixations. The information is perceived by the brain only during fixation. The low-level feature extraction translates the raw eye movements data into fixation and saccades. This reduces the complexity of analyzing eye movement data and retains the cognitive and visual behavior characteristics. In the research work [58], authors have proposed numerous algorithms for fixation identification like I-VT, I-DT, I-HMM, etc. I-VT is the velocity threshold algorithm that separates fixations from saccades based on point-to-point velocities. I-HMM uses Hidden Markov Models to determine fixations, and I-DT is the Dispersion Threshold algorithm that identifies fixations as a group of consecutive eye gaze points within a given dispersion.

**Figure 2:**
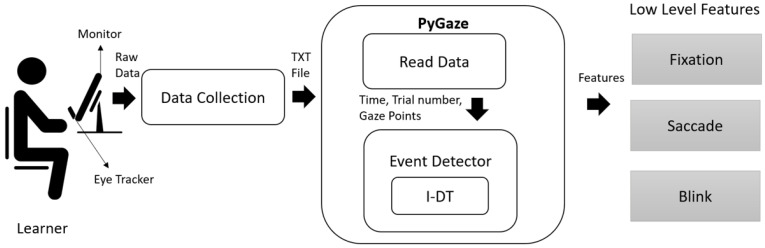
Low Level Feature Extraction.

In our research work, we used the I-DT algorithm for fixation identification. A fixation was identified when eye gaze lasted for at least 100 milliseconds with a maximum dispersion of 100 pixels; else, it was separated as saccades. If the gaze data is zero, then it is marked as a blink. An open source eye tracking toolbox called PyGaze [59] was used for identifying events from the raw eye movements data. The module is schematically represented in the Figure 2. It has two sub modules- Read Data and Event Detector. The *Read Data* module scans the raw eye gaze data and creates a dictionary of information comprising time, trial number, and gaze points. The *Event Detector* uses the I-DT algorithm to identify fixations and saccades.

### 3.4. High Level Feature Extraction

**Figure 3:**
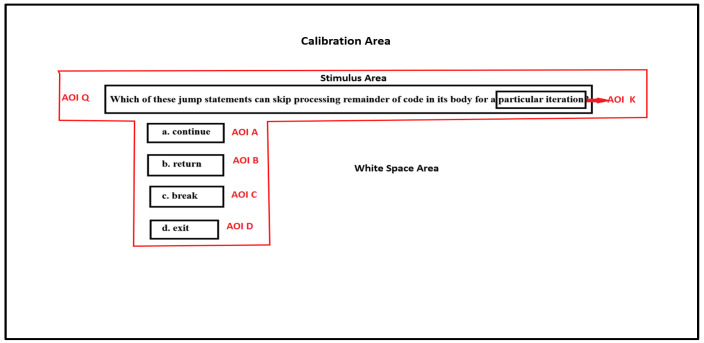
Area of Interest(AOI) representation.

The high level feature extraction module extracts features that indicate various cognitive and visual behavior of the learner. The  admin (using the system for assessment) provides the details of the information of interest to the module as a text file. The information of interest is also known as Area of Interest (AOI). To understand deeper insights into the cognitive behavior of the learners, we identified AOIs in the MCQ. An MCQ has six AOI regions-question (Q), the keyword (K), and four options (A, B, C, D) as represented in Figure 3. The calibration area is the complete screen area. The stimulus area contains the MCQ, and the whitespace is the area with no information. The keyword is the crucial clue in the question that leads to the selection of correct options. The AOI details for extraction of high level features are AOI name, start X and Y pixels, width, and height of the AOI region.

The calibration area is distributed as follows: mean of the correct options (Mean_Correct_ = 14,760 pixels; Mean_Correct_ = 4.09% of stimulus area) was comparable with the incorrect options (Mean_Incorrect_ = 21,271 pixels; Mean_Incorrect_ = 6.05% of the stimulus area). The total stimulus area Mean_Stimulus_ = 41,4962 pixels and Mean_Stimulus_ = 39% of the total screen area. The questions had a Mean_Question_ = 76,386 pixels and Mean_Question_ = 18.19% of the stimulus area. All the fixations outside the stimulus area (whitespace) was excluded from data analysis.

In our research work, we are interested in the information perceived by the learners provided by fixation-related features. Commonly used fixation-related features are fixation duration, fixation count, and time to the first fixation [22,60]. To capture how quickly the information is perceived, the time to first fixation metric is used. Fixation count indicates how many times the learner has viewed the information of interest. Fixation duration indicates how long they have viewed the information of interest.

**Figure 4:**
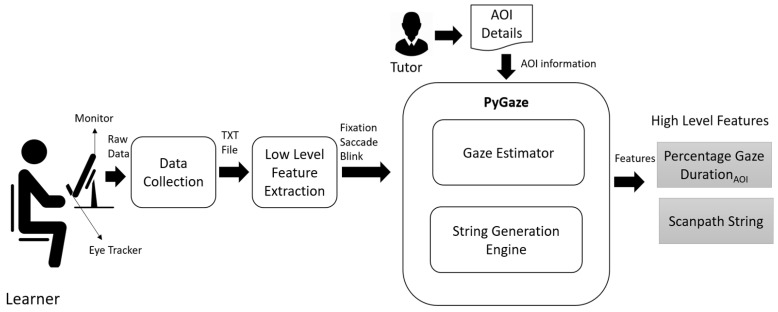
High Level Feature Extraction.

A schematic representation of high level feature extraction module is shown in Figure 4. The high level feature extraction module has two sub modules; Gaze Estimator and String Generation Engine. The *Gaze Estimator* uses the AOI information provided by the tutor to extract fixation-related features at each AOI. The time spent on each information of interest is represented as Percentage Gaze Duration, computed based on Equation (1). The *String Generation Engine* generates a scanpath string based on the sequence in which the learner visited the AOIs. For example, if the learner has gazed at the question, the keyword then options A, B, A, and then C, then the scanpath string is QKABAC. A learner can gaze in the same AOI multiple times; a collapsed scanpath string is generated by eliminating the repeated characters. For example, if the scanpath string of the learner is QQQQQKKABAABCCDAQQ then the collapsed scanpath string is QKABABCDAQ. Scanpath string provides information about how learners processed the information, what is perceived, and what is missed. The scanpath string and percentage gaze duration are the two high level features used in our study for analyzing learner performance.

(1) $$\%GazeDurationAOI_{x}=\frac{FixationDuration_{x}}{\sum FixationDuration}$$

where x = {Q, K, A, B, C, D}.

### 3.5. Data Visualization

Data visualization module helps in the quick analysis of the data. PyGaze provides various methods to visualize the eye movement data like Fixation Map, Heatmap, and Scanpath. Fixation maps are all the fixations of the learner over the stimuli. They are represented using circles. The longer the fixation, the larger the circle. Scanpath is a sequence of fixations and saccades and described using circles and lines or arrows. They provide details of the sequence of gaze over the stimuli. Heatmaps are the most common data visualization that provides the most and least attention of learners over the stimuli.

**Figure 5:**
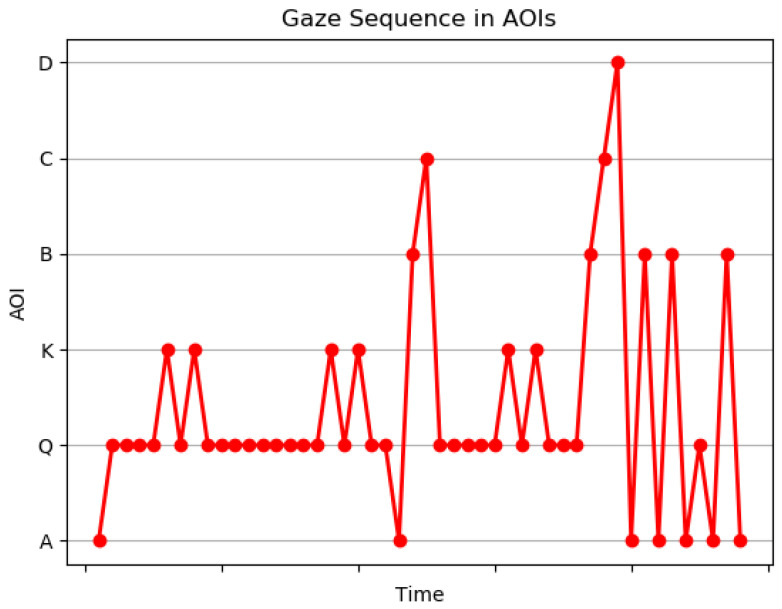
Gaze Sequence Plot.

Scanpath provides only the details of the sequence of fixations and saccades. It does not give the details with context. To interpret the gaze path of the learner, we developed a gaze sequence plot derived from a scanpath very similar to the work in [61]. The gaze sequence plot provides details in terms of the information of interest, as shown in Figure 5. It represents the sequence of AOIs visited over time by the learner.

### 3.6. Intelligent Eye Gaze Learner AsSEssment System-IEyeGASE

The IEyeGASE system provides fine-grained insights into the eye gaze patterns of the learners in an online assessment for assessing their knowledge level at the basic level of Bloom’s Taxonomy called Remember or Recall (Section 4.1). The objective-based online assessment measures the learner’s domain knowledge and how this knowledge leads to choosing the correct option. Eye tracking provides an insight into what is the cognitive process associated with selecting the correct options. The visual and cognitive process of learners during the assessment are indicated by various hypothesis and cognitive parameters like hypothesis of inattentional blindness, wavering behavior, hypothesis of knowledge acquisition, confidence level, engagement and findability. The IEyeGASE system models these cognitive parameters and hypothesis to provide personalized feedback on learner performance. The following subsections describe in detail these cognitive parameters.

#### 3.6.1. Knowledge Acquisition Hypothesis (KA)

**Figure 6:**
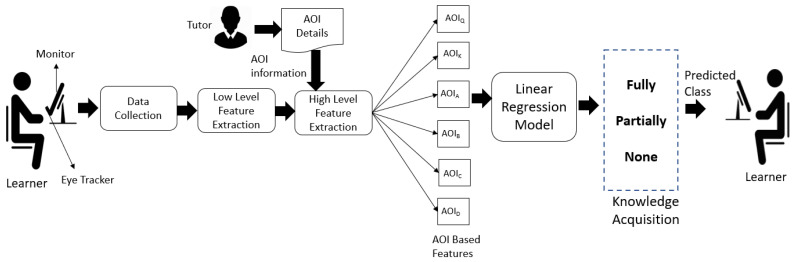
Knowledge Acquisition Model.

Objective-based assessment are used to measure the domain knowledge of the learner. The learners with high MCQ scores have a higher levels of domain knowledge, and learners with low MCQ scores have low domain knowledge. Learners with a high level of knowledge acquisition also called as performing learners, fixate more on correct options than learners with insufficient domain knowledge acquisition called as underperforming learners. This hypothesis investigate the bias to correct the option. It is modeled as a linear regression model represented in Figure 6. The %Gaze Duration on all AOIs are the inputs for model construction, and the actual response (1 = Correct option chosen, 0 = Incorrect option chosen) was used as the input variables. The precise response is collected using the Think Aloud method. The learner thinks aloud the answer(or response) that the observer of the experiment manually records. The predicted values are between 0 and 1 and indicate the level of knowledge acquired by the learner. The predicted values are grouped into three levels, namely None [0–0.3], Partially [0.4–0.5], and Fully [0.6–1]. None means not acquired, Partially means the partial acquisition, and Fully means fully acquired knowledge.

#### 3.6.2. Hypothesis of Inattentional Blindness (IB)

Inattentional blindness or perceptual blindness was observed [62] while conducting experiments on perception and attention experiments. It says that an individuals fails to perceive some part of the stimulus even if they have looked at it. The hypothesis will investigate whether the learner missed important information i.e, keywords, while scanning a question. The tendency to miss the keywords is formulated using the Equation (2). A learner who performs well attend on an average 9% of total gaze duration on keywords. (M_Correct_ = 9.13 SD_Correct_ = 9.36, M_Incorrect_ = 13.55 SD_Incorrect_ = 14.81). The threshold value was set to a lower value 5%. A learner is inattentional blind to keywords when the percentage gaze duration on keyword is less than or equal to 5%.

(2) $$IB=\left\{\begin{array}{cc} YES & \%GazeTime_{k}\leq 5\\ NO & otherwise \end{array}\right.$$

where *k* is keyword and $\%GazeTime{}_{k}$ is the percentage gaze duration on keyword.

#### 3.6.3. Wavering Behavior (WB)

In decision-making literature, subjective preferences are driven by Gaze Bias Effect [4]. Learners will be biased to multiple options and have several revisits before deciding on the correct choice. This characteristic of learners is called Wavering Behavior—the gaze shifts between the most likely options with intermediate visits to questions. Wavering Behavior also leads to confusion and answering incorrect options. It is measured by searching for repeated patterns in the scanpath string. Any repetitive patterns between correct option and incorrect option and question imply a WB. For example, for the stimulus in Figure 3, the correct option is a, and incorrect options are b, c, and d. Thus, a repetitive pattern like ACACAQAC shows a wavering behavior between correct option a and incorrect option c. To identify the wavering behavior of learners, an Algorithm 1 is developed that takes the AOI string and %Gaze Duration on all AOIs as input. Line 1–2 generates the collapsed string as discussed in the high level feature extraction section and generated all repetitive substrings (we only considered the substrings with length 2). The number of times the substrings are repeated is computed. In line 3 the maximum repeated substring is identified. Line 4–6 computes the time spent by the learner while gazing between two options in MCQ computed as the sum of all combinations of AOIs. The AOIs of the maximum computed value are identified. This provides information about the two options where the learner has gazed maximum. Line 7 checks if a wavering behavior is observed in the learner. This is based on three conditions:

1. If the correct option is in the maximum repeated substring
2. If the correct option is the AOIs where the learner has spent maximum time.
3. If the AOIs with maximum sum is equal to the most repeated substring

The learner may also have repeated gaze among two incorrect options; in such cases, we assume that the learner’s knowledge in the concept is poor.
**Algorithm 1:** Algorithm for Wavering Behavior**Result**: Wavering Behavior **Input**: AOIString, %Gaze Duration on each AOI, CorrectOption Generate collapsed stringGenerate all repetitive substrings and compute their repetitive countsIdentify the maximum repeated substringFor all maximum combinations of AOIs compute the sumIdentify maximum sum from all combinationsIdentify the AOI combination with maximum sumWavering Behavior is observed if the following conditions are met
(a)CorrectOption in the maximum repeated string(b)Correct Option in AOI combination with maximum sum(c)AOI combination with maximum sum equals maximum repeated substring

#### 3.6.4. Learner Confidence Level Hypothesis

Learner competence is mostly inferred by the correct percentage score obtained in an MCQ assessment. The lack of confidence in the correct option is seldom evaluated. Learners may have gaze bias to the correct option but finally chooses the wrong choice. Eye tracking technology can provide an insight into the confidence level of learners on correct options. The learner may gaze at the correct option for a long time but end up choosing the incorrect option. The hypothesis is measured by measuring the fixation duration on the correct option versus the incorrect option. For example, a learner must have gazed at the correct option a in Figure 3, but end up choosing option c. This can be due to the low confidence in the correct answer.

#### 3.6.5. Findability and Engagement

Other Cognitive parameters of interest are findability and engagement. Findability is the quickness in selecting the correct option. It is computed based on the response time of the learner. Engagement is the time spent by the learner in completing the task. The stimuli have two parts, the Stimulus area, and Whitespace area, as represented in Figure 3. For example, if the learner spends 80% of total gaze time on the Stimulus area, the learner is engaged the task.

## 4. Experimental Design

This section describes the experimental design for developing the intelligent system like Bloom’s Taxonomy, experiment procedure, apparatus, participants details etc.

### 4.1. Bloom’s Taxonomy

Bloom’s Taxonomy is a hierarchical way of classifying different levels of thinking and applied to design course objectives. The course objectives provide details of expectations from learners during the end of the course [36,63]. Bloom’s Taxonomy proposes six levels of thinking-Remember, Understand, Apply, Analyze, Evaluate, Create.

In [63], authors describe various levels of Bloom’s Taxonomy to differentiate between cognitive skill levels and how these levels can lead to deeper learning and transfer of knowledge and skills to a greater variety of tasks and contexts. Remember, the first level of thinking leads to developing skills crucial for completing the pedagogical process. It is about recalling the concepts from memory. Understand is the second level where the learner explains ideas and concepts, discusses and describes them, and translates the points somehow. In the Apply level, the information learned is applied in new situations to solve a problem. Critical thinking happens in the Analyze level, where they can distinguish between fact and opinions and breaks down the information into smaller components. In the Evaluate state, the learner justifies a decision through thoughts based on the knowledge acquired. The topmost level is Create, where learners produce new creative ideas of their own in solving problems. This framework provides the ability to tutors to create achievable learning goals and help develop plans to meet them. It ensures learners demonstrate the cognitive skills in solving each problem. Evaluators can apply this taxonomy by asking questions in the form of MCQs to correlate with specific learning goals at the basic level of Remember and Understand.

In our research work, the focus is on the basic level of thinking; Remember, this level is crucial for laying a solid foundation for learning. At this level, learners memorize the facts and concepts and recall when required. All the materials used in the study were discussed with the tutor, and MCQs were defined to achieve the goal of understanding the basic level of learning, Remember.

### 4.2. Materials

The objective-based assessment used in the present study consisted of five MCQs based on Object-Oriented Programming with Java [64]. Since the objective is to test the lower level of Bloom’s Taxonomy, topics include keywords, operators, decision-making, loops, constructors, object creation, and initialization in Java. All the MCQs are oriented towards recall from long-term memory. This means, no logical or problem-solving questions were tested. Each MCQ is a short question with a keyword that can provide a clue to the correct option. Each MCQ comprises four alternatives, namely the correct choice and incorrect options that are distractors. The correct option implies that the concept is learned. The distractors are closely associated with the correct option and creates confusion in learners’ minds. The order of the presentation of options is not of interest in the design of the present study. The following are the MCQs with keywords that provides valuable information towards the correct choice and various answer options:

- **MCQ1**: Which keyword is used by method to refer to the object that invoked it?
  **Keyword**: object that invoked it
  **Options**
  1. import (Incorrect)
  2. this (Correct)
  3. catch (Incorrect)
  4. super (Incorrect)
  **Concept Learned**: Object creation and initialization
- **MCQ2**: List the arithmetic operators in increasing order of precedence.
  **Keyword**: increasing order of precedence
  **Options**
  * + % - / (Incorrect)
  * / - + % (Incorrect)
  * / % + - (Correct)
  * % / + - (Incorrect)
  **Concept Learned**: Operators in Java
- **MCQ3**: Which of the following are keywords in Java?
  **Keyword**: keywords
  **Options**
  1. while, switch, if, static, bool (Incorrect)
  2. while, switch, Boolean, static, pack(Incorrect)
  3. break, catch, ball, return, switch (Incorrect)
  4. while, switch, break, Boolean, catch (Correct)
  **Concept Learned**: Keywords in Java
- **MCQ4**: Which keyword is used to invoke base class constructor?
  **Keyword**: invoke base class constructor
  **Options**
  1. this (Incorrect)
  2. import (Incorrect)
  3. refer (Incorrect)
  4. super (Correct)
  **Concept Learned**: Constructor
- **MCQ5**: Which of these jump statements can skip processing remainder of code in its body for a particular iteration?
  **Keyword**:particular iteration
  **Options**
  1. continue (Correct)
  2. return (Incorrect)
  3. break (Incorrect)
  4. exit (Incorrect)
  **Concept Learned**: Decision Making and Loops

### 4.3. Participants

Fifteen learners from the Amrita Vishwa Vidyapeetham University, Bengaluru, India volunteered for the experiment. The learners comprised graduates from the department of computer science and engineering undergoing a course in Object-Oriented programming using Java. The learners either had a prior knowledge of C and C++ programming or no prior programming knowledge. There were three female and twelve male participants, and their average age was 19 years. The learners had normal or corrected vision. The participants were detailed on the study procedure and intimated that they could abandon the study anytime. The total samples collected are 75 (15 learners and 5 MCQs).

### 4.4. Apparatus

Eye movements were recorded using a remote, video-based Senso Motoric Instruments (SMI) REDn Professional eye tracker [65], with frequency 60 Hz. The study was conducted in the eye tracking lab and learners were tested individually. The screen resolution is 1366 × 768 pixels. The experiment was set up using the software SMI Experimental Suite 360°. Each MCQ were presented on a single screen. The learners were seated at a distance of 60–70 cm from the monitor. The eye tracking system was calibrated on a 9-point calibration image. In case, the calibration accuracy is below 0.7 degrees of visual angle, the observer requested recalibration.

### 4.5. Procedure

The experimental study was conducted in the eye tracking lab at Amrita Vishwa Vidyapeetham university. Time slots were provided to the volunteers of the study. After signing informed consent, learners were briefed on the do’s and dont’s of the eye tracking study. Next the eye tracking equipment was calibrated and after successful calibration, learners were once more reminded to think aloud the answers and the begin the assessment. The learners worked at their own pace. There was no option for moving to previous MCQ and to move to next question, they pressed enter key. All the MCQs were shown in the same order to all learners. Learners were rewarded with a small treat [fruit juice and chocolate].

### 4.6. Data Analyses

The test score of the learner were manually recorded in a spread sheet. All the statistical analysis were conducted in R 3.5 version. PyGaze analyzer was used to detect low level features and high level features of the learner system. The intelligent IEyeGASE system was developed in Python 3.7.

## 5. Results

The IEyeGASE system provides deeper insights into the learner’s performance for each question in the objective-based assessment. The results of each hypothesis are discussed in this section. Table 1 shows the list of learners who answered the questions correctly (performing learners) and incorrectly (underperforming learners) based on the response for each MCQs. The tags S1, S2, etc., represent the learner ID. The table represents learners performance for each MCQs. The column *Correct Option* is the answer to the MCQs discussed in the Section 4.2. The columns *Learners Answered Correctly* and *Learners Answered Incorrectly* list the learner ID of learners who got the MCQ right and wrong, respectively. Each question aims at evaluating different concepts discussed in the material section. The following subsection discusses the results of the IEyeGASE system.

### 5.1. Hypothesis of Inattentional Blindness (IB)

**Figure 7:**
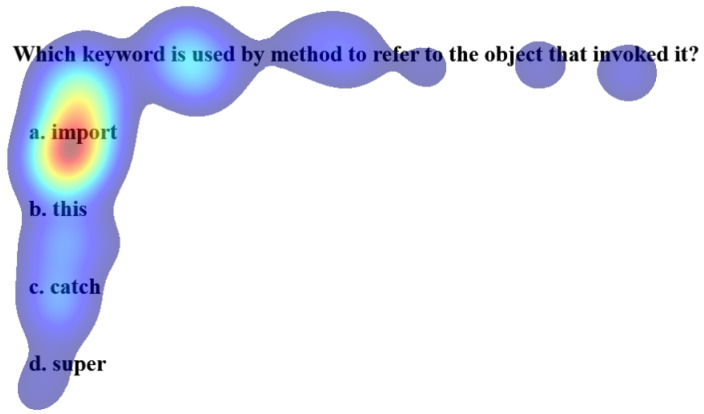
Heatmap of learner S6 on MCQ1 representing Inattentional Blindness.

**Figure 8:**
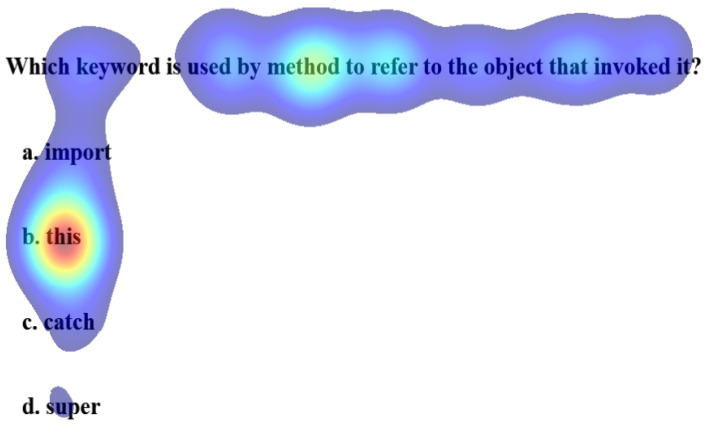
Heatmap of learner S1 on MCQ1 representing no Inattentional Blindness.More gaze on keyword and correct option.

The Welch two-sample t-test reveals no significant differences in the gaze data on keywords and correctly choosing the option for performing and underperforming learners. Table 2 shows the results of the test. However, a paired t-test between the percentage gaze duration on keyword and correct option shows significant differences for MCQ2, MCQ3, and MCQ4 as represented in Table 3. It implies that gazing at keywords leads to the correct option. The heat maps in Figure 7 and Figure 8 distinguish the inattention blindness of an underperforming learner and performing learners. Figure 7 shows that the fixation of an underperforming learner S6, on the keywords “object that invoked it” is low, and the option chosen by the learner was an incorrect option *a import*. This indicates that the learner failed to perform as the critical clue in the question was missed. Figure 8 shows that the fixation on performing learner S1, on the keywords “object that invoked it” was high and that leads to the fixation on the correct option *b. this*. The statistical analysis and the visualizations clearly show that gazing at important clues, leads to selecting the correct option. The IEyeGASE system predicted 36% of the learners had inattentional blindness. 16% of the learners answered the question wrong due to inattentional blindness.

### 5.2. Wavering Behaviour

**Figure 9:**
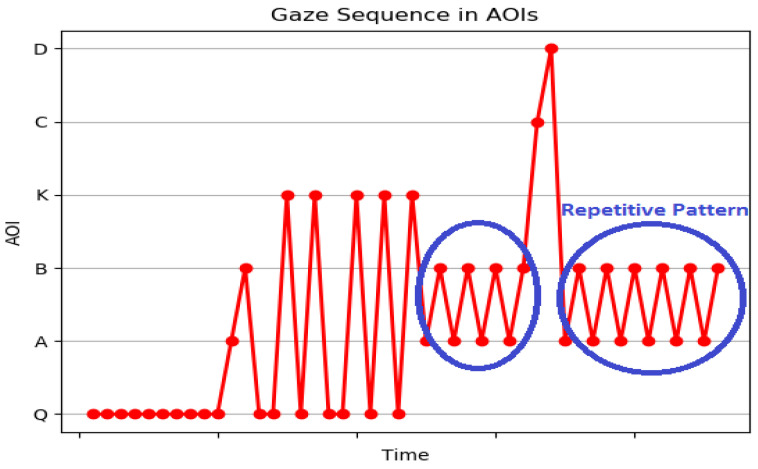
Gaze sequence plot representing WB of a underperforming learner where WB is observed between incorrect options.

**Figure 10:**
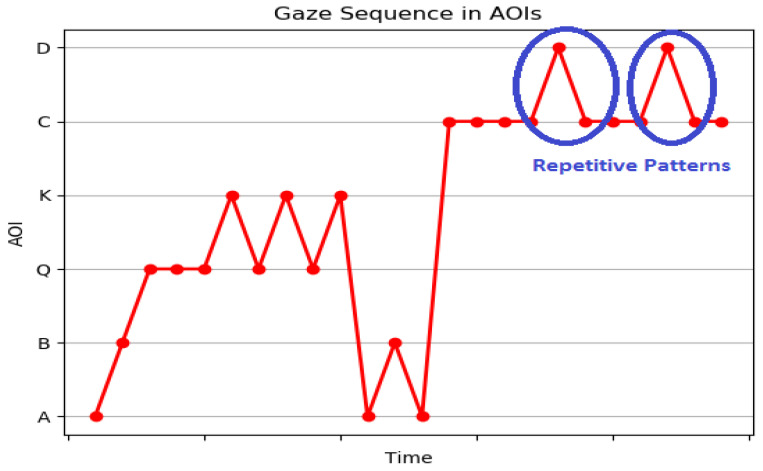
Gaze sequence plot representing WB of a performing learner where WB is observed between correct and incorrect options.

A wavering behavior is observed when learners are not sure which option to choose, and they have a repetitive gaze between the different options. The alternative options in MCQs are distractors introduced to investigate confusions in the mind of learners. Figure 9 demonstrates the gaze wavering of an underperforming learner between options a and option b for MCQ1. The correct option is option b. The learner ended up in selecting the incorrect option. The results indicate confusion of options in underperforming learners. A wavering behavior was observed in performing learner for MCQ 2 between incorrect option d and correct option c as shown in Figure 10. The gaze duration was more on option c, and the learner ended up selecting the correct option. This can also be a mere guess. Based on the %Gaze duration and AOI string, the IEyeGASE system predicted wavering behavior in 17% learners and 8% of them failed to perform, and 9% of them guessed the correct answer.

### 5.3. Knowledge Acquisition Hypothesis

Table 4 represents the number of samples of learners performing and underperforming categorized in different classes of KA. The IEyeGASE linear regression model predicts the knowledge acquisition of learners as Fully, Partially, and None. Considering Fully and None classes, the regression model produces an accuracy of 76% when compared to the ground truth response value. When further analyzed, out of the nine underperforming learners where the model predicted as Fully acquired, we observed three learners with WB; three learners lacked confidence in answering the correct option, one learner with less engagement on stimuli, and others unknown reason. Similarly, while analyzing the eye gaze data of 6 performing learners, the IEyeGASE linear regression model predicted as None acquired, all the learners were quick at task and hence the time spent on the correct option was less. The underperforming learners who partially acquired knowledge, one learner had WB, two learners did not engage in the task, one learner with inattentional blindness, and one learner lacked confidence. These insights were provided to the tutor for further validation and feedback to learners for improvement.

### 5.4. Findability and Engagement

**Figure 11:**
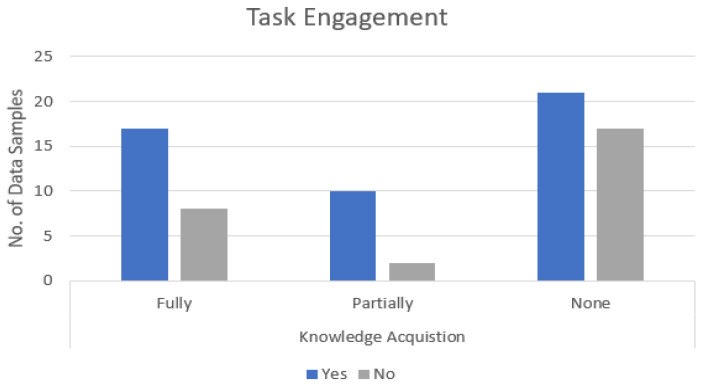
Graph representing engagement in task for learners in different KA classes.

The findability and engagement of the learners in each task were analyzed. The IEyeGASE system predicted learners S3, S5, S9 and S14 quick at different tasks. S3 was fast at MCQs 1 and 3, S5 at MCQs 3 and 4 and S9 at MCQ 3. 26 learner samples were predicted not engaged in the task. The learners were either quick at the task or not interested in the task or gazed at whitespaces than the stimulus area Figure 11 represents the engagement (YES and NO) of learners in different KA classes. The graph shows that to complete a task, engagement is an essential factor. We also found that learners who are categorized as KA None too were engaged in the task. They might be trying to recollect the answers from memory.

### 5.5. Confidence Level Hypothesis (CL)

**Figure 12:**
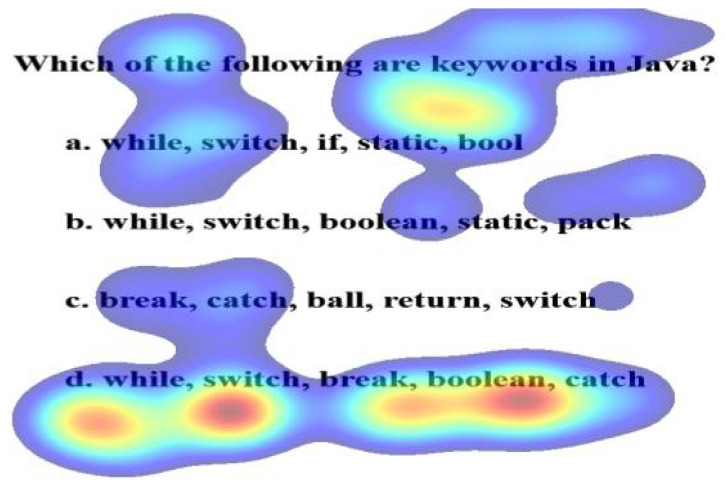
Heat map representing Lack of Confidence by an underperforming learner for MCQ3.

The learners can have gaze biased to the correct option but chooses an incorrect option. Figure 12 represents a heat map of an underperforming learner whose maximum gaze is on option D, which is a correct option but fails to answer. This implies that to choose the correct option, the learner should know and the confidence to select it. The IEyeGASE system predicted three learners exhibiting a lack of confidence S8, S13, and S15. S8 on MCQ 2, S13 on MCQs 3 and 5, and S15 on MCQ 2.

## 6. Discussion

IEyeGASE system offers similar functionalities present in an online assessment system. However, several features differentiate it from other assessment systems. A comparison with traditional pen and paper assessment and online assessment platforms can be seen in Table 5. All assessment system provides a score that quantifies the performance of the learner. The online assessment system and IEyeGASE system provides an easy to use interface to visualize the personalized results. In addition, both these systems do not require a dedicated resource like a tutor or evaluator to interpret the results. Response time and engagement in the task in the case of online assessment are based on how quick and how long it took to select an option. In the case of the IEyeGASE system, it is based on how quickly the learner gazed at the option and how long on the AOIs.

**Figure 13:**
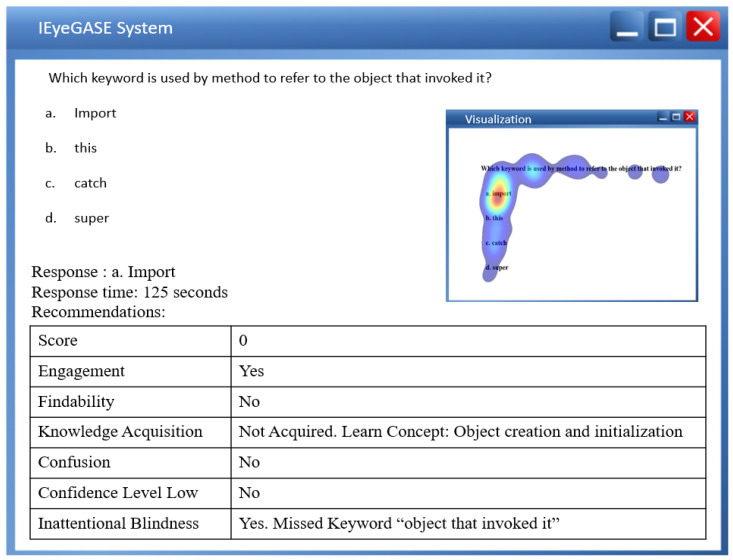
Personalized feedback of a poor learner.

**Figure 14:**
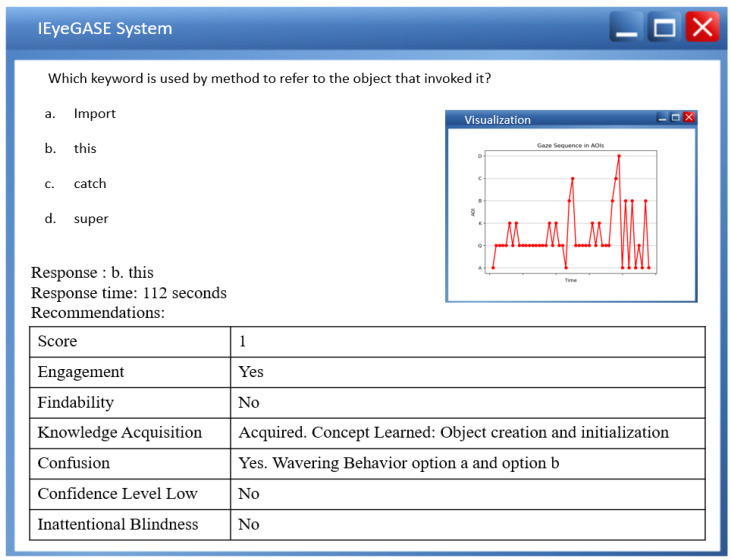
Personalized feedback of a good learner exhibiting wavering behavior.

The IEyeGASE system provides a personalized feedback on the performance of the learner. Figure 13 shows personalized feedback of a poor learner for the concept “Object creation and initialization”. The learner missed the information “object that invoked it” and hence failed to answer the correct option. Figure 14 shows the personalized feedback of a good learner also exhibiting a wavering behavior between options *a. import* and *b. this*. The visualization shows the learner gazed at options a and b multiple times while also gazing at keyword and question. The personalized feedback helps the learner and the evaluator get better insights into the performance even though the score reveals the learner as a good learner.

Table 6 summarizes the more profound insights into the various aspects of learners for each MCQ. The traditional pen and paper approach or online assessment fails to bring these insights into learners’ performance. The IEyeGASE system provides detailed feedback on learners’ performance that can be used by tutor to improve the quality of education and redefine the learner goals. For example, learner S1 performed well for MCQs 1, 2, 3 and 5. A wavering behavior was observed while answering MCQs 4 and 5. The wavering behavior resulted in not performing for MCQ 4. In case of MCQ 5, the learner was confused between options, this indicate that the learner needs to redo the concept. In another example, learner S6 was lucky enough to answer the MCQs 3 and 5. However, the learner did not perform for other MCQs and ignored critical information in the questions. The engagement in the task was poor for MCQs 3, 4 and 5.

The learners S1, S3, and S11 were females, and others were males. The study [66] investigates the difference in viewing of male and female participants. The results indicate females showed more exploratory gaze behavior and short fixation ratios. A quick analysis of the results of the IEyeGASE system indicates a similar behavior in female learners. They were primarily engaged in the task and especially found S3 quick with short fixations on options. Male learners, especially S8, S13 and S15, exhibited low confidence in answering the correct option. An extensive analysis of the differences in learner behavior of female and male learners is not within the scope of the present study.

The tutor can use the insights from the personalized feedback to understand why the learner failed to perform or what factors affected their performance while trying to recall information from memory. The present system focused on predicting learners’ performance in the lower level of Bloom’s Taxonomy. We plan to extend the work in understanding the parameters affecting critical thinking in the higher levels of Bloom’s Taxonomy.

### Limitations

The limitation of the IEyeGASE system is that the system provides personalized assessment for independent tasks and does not provide continuous monitoring of learner’s progress. Instead, the system provides feedback to tutors and learners to help them recalibrate their learning goals. Another limitation is, the system only works with objective assessment. However, cognitive parameters like engagement and inattentional blindness can be extended to subjective assessments.

The current IEyeGASE system does not provide insights into cognitive parameters related to higher-level thinking of Bloom’s Taxonomy. However, the parameters discussed are also applicable for other levels. Further studies will incorporate specific cognitive parameters that are indicators for higher levels of thinking.

Data analysis is limited to eye gaze data; the other data sources like the audio, mouse, video are not considered.

The present study is conducted on a limited number of learners and will be extended to a large number of learners.

## 7. Conclusions

The assessment system has mostly moved from the traditional pen and paper to objective-based online assessment. The conventional methods of evaluation provide feedback on performance based on the score obtained. What is perceived and what affected the performance of learners is crucial for tutors to plan for lessons. An intelligent learner assessment system called IEyeGASE was developed; it uses eye gaze data to quantify the factors affecting the learner’s performance. The IEyeGASE system evaluates the learner on the low level of Bloom’s Taxonomy, Remembrance. The various models of the IEyeGASE system provide feedback based on the cognitive parameters measured like engaged in a task, quickness, knowledge acquisition, confidence level, inattentional blindness, and wavering behaviour. The learner can use the feedback to learn better and tutors to improve the quality of learning and redefine the learning goals. The IEyeGASE system can be easily integrated into existing digital evaluation systems to provide deeper insights into the learner’s performance. In the future, the intelligent assessment system will be extended to understand and quantify the cognitive parameters affecting critical thinking in the higher levels of Bloom’s Taxonomy.

## Figures and Tables

**Table 1:** Learner response for each MCQ.

MCQ	Correct Option	Learners Answered Correctly	Learners Answered Incorrectly
MCQ1	B	S1, S3, S4, S5, S7, S10, S11, S12, S13, S14	S2, S6, S8, S9, S15
MCQ2	C	S1, S3, S4, S5, S9, S12	S2, S6, S7, S8, S10, S11, S13, S14, S15
MCQ3	D	S1, S3, S5, S9, S12, S14	S2, S4, S6, S7, S8, S10, S13, S15
MCQ4	D	S5	S1, S2, S3, S4, S6, S7, S8, S9, S10, S11, S12, S14, S15
MCQ5	A	S1, S3, S5, S8, S10, S12, S14	S2, S4, S6, S7, S11, S14, S15

**Table 2:** Mean and Standard Deviation of performing and underperforming learners for %Gaze Duration on keywords.

MCQ	Performing Learners N, M(SD)	Underperforming Learners N, M(SD)	*p*-Value
MCQ1	10, 15.1(7.44)	5, 8.6(7.68)	0.141
MCQ2	6, 6.83(8.00)	9, 10.30(4.17)	0.288
MCQ3	6, 2.3 (2.6)	9, 1.9 (2.14)	0.754
MCQ4	1, 36.8(NA)	14, 29.9(16.03)	0.683
MCQ5	7, 4.45(4.01)	8, 4.68(3.37)	0.907

N = sample, M = Mean, SD = Standard Deviation.

**Table 3:** Mean and Standard deviation of gazing at correct option and keyword.

MCQ	*t* Value(14)	*p*-Value
MCQ1	0.40362	0.6926
MCQ2	2.9576	0.01
MCQ3	3.24	0.0059
MCQ4	−5.925	0.00003
MCQ5	1.9	0.07

**Table 4:** Knowledge Acquisition in learners.

Knowledge Acquisition	Performing	Underperforming
Fully	17	9
Partially	7	6
None	6	30

**Table 5:** Comparison between different assessment techniques.

Parameters	Traditional Assessment	Online Assessment	IEyeGASE
Score	Yes	Yes	Yes
User Interface	No	Yes	Yes
Additional Infrastructure	No	Yes	Yes
Dedicated Resource	Yes	No	No
Personalized Feedback	No	Yes	Yes
Response Time	No	Yes	Yes
Visualization of Results	No	Yes	Yes
Knowledge Acquired	No	Yes	Yes
Wavering Behavior	No	No	Yes
Confusion in mind	No	No	Yes
Inattentional Blindness on Keywords	No	No	Yes
Engagement in Task	No	Yes	Yes

**Table 6:** Summary of Deeper Insights into each learner’s performance.

Learner ID	MCQ 1	MCQ 2	MCQ 3	MCQ 4	MCQ 5
S1	KA(F)	KA(F)	KA(F)	KA(N), WB	KA(F), WB
S2	KA(P), WB	KA(N), IB, E(N)	KA(N)	KA(N)	KA(F)
S3	KA(N), F(Y)	KA(F), WB, IB	KA(N), IB, F(Y)	KA(N), WB	KA(F), IB
S4	KA(P), WB	KA(P)	KA(N), IB	KA(N)	KA(F), WB
S5	KA(F), WB	KA(F)	KA(N), IB, F(Y), E(N)	KA(N), F(Y)	KA(F)
S6	KA(N), IB	KA(P), E(N)	KA(F), WB, IB	KA(N), E(N)	KA(F), WB, IB, E(N)
S7	KA(P), WB, IB	KA(N)	KA(P), IB	KA(N)	KA(P)
S8	KA(N)	KA(F), CL(Y), E(N)	KA(N), IB	KA(P)	KA(F), IB
S9	KA(N), IB, E(N)	KA(F), IB, E(N)	KA(N), IB, F(Y), E(N)	KA(N), IB, E(N)	KA(N), IB, E(N)
S10	KA(F)	KA(N)	KA(N), E(N)	KA(N)	KA(P)
S11	KA(P)	KA(N)	KA(N), E(N), IB	KA(N)	KA(F), IB
S12	KA(F), E(N)	KA(F)	KA(F)	KA(N), E(N)	KA(F), IB
S13	KA(F), WB, E(N)	KA(N), E(N)	KA(F), IB, F(Y), E(N)	KA(N), E(N)	KA(P), CL(Y), E(N)
S14	KA(P), WB	KA(N), E(N)	KA(N), IB, F(Y), E(N)	KA(N), E(N)	KA(F), IB, E(N)
S15	KA(N)	KA(F), CL(Y), E(N)	KA(N), IB	KA(N)	KA(F), IB, E(N)

KA = Knowledge Acquisition (F = Fully, P = Partially, N = None), IB = Inattentional Blindness, WB = Wavering Behavior, F(Y) = Findability(Yes), E(N) = Engagement(No), CL(Y) = Low Confidence Level (Yes).

## Data Availability

Not Applicable.
